# The genotype and phenotype of chromosome 18p deletion syndrome: Case series

**DOI:** 10.1097/MD.0000000000025777

**Published:** 2021-05-07

**Authors:** Qiujie Jin, Rong Qiang, Bo Cai, Xiaobin Wang, Na Cai, Shuai Zhen, Wen Zhai

**Affiliations:** Center of Medical Genetics, Northwest Women's and Children's Hospital, Xi’an, Shaanxi, PR China.

**Keywords:** array-based comparative genomic hybridization, chromosome 18p deletion syndrome, karyotype analysis, phenotypes, prenatal diagnosis

## Abstract

**Rationale:**

The chromosome 18p deletion syndrome is a syndrome with a deletion of all or a portion of the short arm of the chromosome 18. The phenotypes of the chromosome 18p deletion syndrome vary widely among individuals due to differences in size and breakpoints and the involved genes on the deletions. Given the varied and untypical clinical presentation of this syndrome, the prenatal diagnosis of the syndrome still presents as a challenge.

**Patient concerns:**

We described 4 China cases with different chromosomal breakpoints. In case 1, a woman who with mild phenotypes gave birth to a severely deformed fetus. Three other cases were for prenatal diagnosis. Their phenotypes are the increased nuchal translucency (INT) and the noninvasive prenatal testing (NIPT) indicated deletions on the chromosome 18p and severe hydronephrosis respectively.

**Diagnosis:**

The 4 cases were diagnosed with chromosome 18p deletion syndrome through karyotype analysis and array-based comparative genomic hybridization (array-CGH).

**Interventions:**

Karyotype analysis and array-based comparative genomic hybridization were used to analyze the abnormal chromosome.

**Outcomes:**

Case 1 and case 2 revealed 11.51 and 12.39 Mb deletions in 18p11.32p11.21. Case 3 revealed 7.1 Mb deletions in 18p11.3218p11.23. Case 4 revealed 9.9 Mb deletions in 18p11.3218p11.22.

**Lessons:**

In our report, we are the first to report that mother and progeny who have the same chromosomal breakpoint have different phenotypes, significantly. In addition, we found a new phenotype of chromosome 18p deletion syndrome in fetus, which can enrich the phenotypes of this syndrome in the prenatal diagnosis. Finally, we demonstrate that the individuals with different chromosomal breakpoints of 18p deletion syndrome have different phenotypes. On the other hand, the individuals with the same chromosomal breakpoints of 18p deletion syndrome may also have remarkably different phenotypes.

## 1 Introduction

The chromosome 18p deletion syndrome (Online Mendelian Inheritance in Man [OMIM] #146390) is a contiguous gene deletion syndrome that results from the deletion of all or a portion of the short arm of chromosome 18. The incidence of the chromosome 18p deletion syndrome is estimated at 1 in every 50,000 live births, and the female to male ratio is 3:2.^[1,2]^ Given the different sizes of missing fragments and specific breakpoints, the clinical phenotypes are broad.^[3]^ The main clinical symptoms involve short stature, mental retardation, special features, dystonia, genitourinary system dysplasia, and brain malformation.^[2,4–6]^

Although chromosome 18p deletion syndrome can affects the patients’ quality of life and survival in different degrees, but no specific treatment is available. Therefore, the prenatal diagnosis of the 18p deletion syndrome is important. However, given the varied and untypical clinical presentation of this syndrome, the prenatal diagnosis of the syndrome still presents as a challenge.

In our report, we found 4 China cases with the chromosome 18p deletion syndrome by using karyotype analysis and array-based comparative genomic hybridization (array-CGH). Through our report, we hope to provide some assistance for prenatal diagnosis and genetic counseling of the chromosome 18p deletion syndrome.

## 2 Case presentation

### 2.1 Case 1: Abnormal pregnancy history case

A 27-year-old woman with adverse pregnancy history came to our center for genetic counseling. A review of her medical history revealed that she had terminated her pregnancy at 24 weeks 1 year ago due to holoprosencephaly. The fetal ultrasound diagnosis showed an abnormal cerebellar morphology, a single ventricle, unclear cavum septi pellucidi and brain middle, significantly narrowed intraorbital distance, and a single-nostril nose. The CNV-Seq results of the fetus were seq[hg19]18p11.32p11.21(120001-11580000)×1.

The woman was 159 cm tall with a round face and a thick neck. She had mild language development delay. She can communicate with people normally and live on her own. The woman graduated from a junior college. Currently, she worked full time in the restaurant business. The woman had no family history of genetic disease. Her brain magnetic resonance imaging scan showed a normal brain structure. Thus, the karyotype and chromosome microarray analyses were suggested.

### 2.2 Three prenatal cases

#### 2.2.1 Case 2

A 40-year-old pregnant woman with a single fetus, 2 pregnancies, 1 parturition, and gestational age of 26 weeks and 3 days had given birth to a healthy daughter. She and her husband were healthy and had no family history of genetic disease. The woman came to our center for genetic counseling due to the fetus with a severe hydronephrosis in the left kidney. The fetal ultrasound examination suggested that the size of the left kidney was 47 mm × 31 mm × 22 mm, and the collecting system of the renal pelvis was separated. The anteroposterior diameter was 17 mm, and the widest part was 4 mm. The size of the right kidney was 32 mm × 21 mm × 17 mm. Therefore, she underwent amniocentesis to perform fetal amniotic fluid karyotype and chromosome microarray analyses.

#### 2.2.2 Case 3

A 28-year-old G2P1 woman with normal ultrasound findings during the whole pregnancy period came to our center. She had no family history of genetic disease and had given birth to a healthy daughter. Thus, the noninvasive prenatal testing (NIPT) was performed to screen for fetal chromosomal abnormalities. Results revealed 7.1 Mb deletions from 18p11.32 to 18p11.23. Therefore, the amniotic fluid was extracted using amniocentesis at 22 weeks and 3 days of gestation. Chromosomal karyotypes and chromosomal microarray analyses were detected in amniotic fluid cells.

#### 2.2.3 Case 4

A 31-year-old G2P1 woman underwent amniocentesis at 19 + 2 weeks’ gestation because of increased nuchal translucency (INT, 3.5 mm) at 12 + 2 weeks’ gestation. The woman and her husband were healthy and had no family history of genetic disease. She had given birth to a healthy son. Fetal cells were extracted from the amniotic fluid for analysis of the fetal karyotype and the chromosome microarray analyses.

## 3 Methods

### 3.1 Karyotype analysis

The peripheral blood was collected in heparin anticoagulant tubes. The amniocentesis was performed under the guidance of ultrasound, and the amniotic fluid was collected. The blood cells or fetal amniotic fluid exfoliated cells were cultured and G-banded in accordance with the standard protocols. The chromosomal karyotypes were scanned and analyzed using the automatic chromosome scanning analysis system (MetaSystems, ZEISS, Oberkochen, Baden-Wurttemberg, Germany).

### 3.2 Array-based comparative genomic hybridization (array-CGH)

The collected peripheral blood in the ethylene diamine tetraacetic acid anticoagulant tubes and amniotic fluid was obtained using amniocentesis. The genomic DNA was extracted from the blood and the amniotic fluid. The test genomic DNA and sex-matched human reference DNA were labeled with different fluorophores. Equal quantities of the 2 labeled DNA samples were mixed and cohybridized to a 8 × 60 K commercial array (Agilent Technologies, Santa Clara, California, United States). The chip was scanned using the SureScan Microarray Scanner (Agilent Technologies), and the Cytogenomics software (Agilent Technologies) was used to analyze the results. Finally, we used the Genoglyphix (PerkinElmer, Waltham, MA,United States) to analyze the pathogenicity of the mutant fragment online.

The study was approved by the ethics committee of Northwestern Women's and Children's Hospital. All of the tests were performed under the patient's consent and the patients have given written informed consent.

## 4 Results

### 4.1 Case 1: Abnormal pregnancy history case

**Figure 1:**
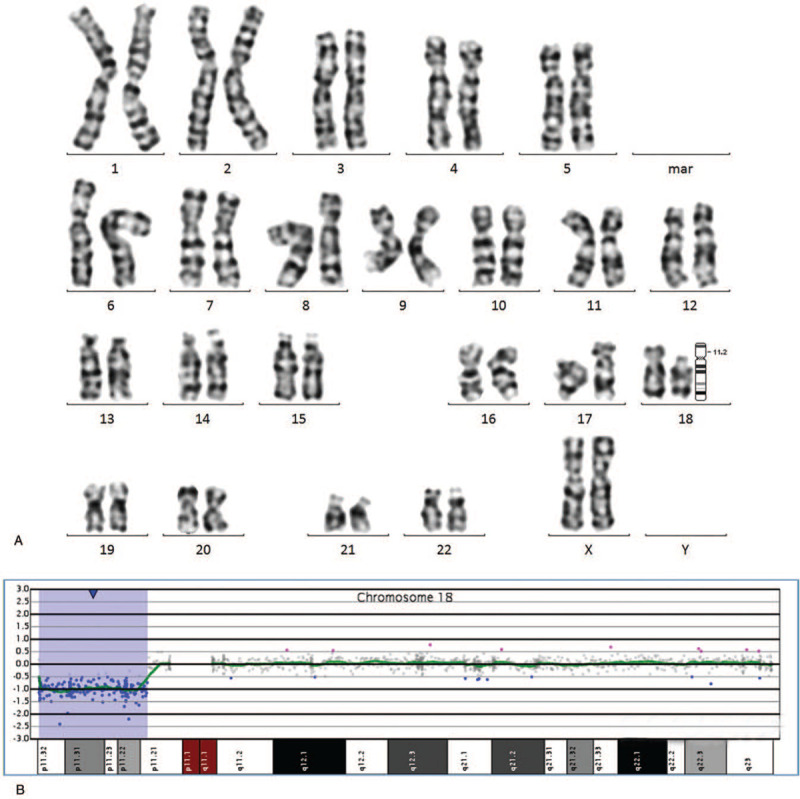
The results of karyotype analysis and array-CGH in case 1. (A) The results of karyotype analysis: 46, XX, del (18)(p11.2). (B) The results of array-CGH:arr[GRCh37]18p11.32p11.21 (146484_11654499)×1.

The chromosome karyotype revealed that the woman had an abnormal karyotype of 46, XX, del (18)(p11.2) (Fig. 1A), and the array-CGH revealed 11.51 Mb deletion in arr[GRCh37] 18p11.32p11.21(146484_11654499) (Fig. 1B). It contains 38 OMIM genes, including *USP14*, *THOC1*, *COLEC12*, *CETN1*, *CLUL1*, *TYMS*, *ENOSF1*, *YES1*, *ADCYAP1*, *NDC80*, *SMCHD1*, *EMILIN2*, *LPIN2*, *MYOM1*, *MYL12B*, *TGIF1*, *DLGAP1*, *AKAIN1*, *ZBTB14*, *EPB41L3*, *L3MBTL4*, *ARHGAP28*, *LAMA1*, *PTPRM*, *RAB12*, *GACAT2,MTCL1*, *NDUFV2*, *ANKRD12*, *TWSG1*, *RALBP1*, *PPP4R1*, *RAB31*, *TXNDC2*, *VAPA*, *APCDD1*, *NAPG*, and *PIEZO2*. We all know that the location of the missing sections had subtle differences due to different detection methods. Thus, the woman and her fetus had slightly different chromosome localizations of the deletions. Then, blood samples were obtained from her parents to perform further karyotyping, and results showed that her parents had no chromosome abnormality. Results proved that the deletion in the woman was a de novo deletion, but the woman transmitted the deletion to her fetus.

### 4.2 Three prenatal cases

#### 4.2.1 Case 2

**Figure 2:**
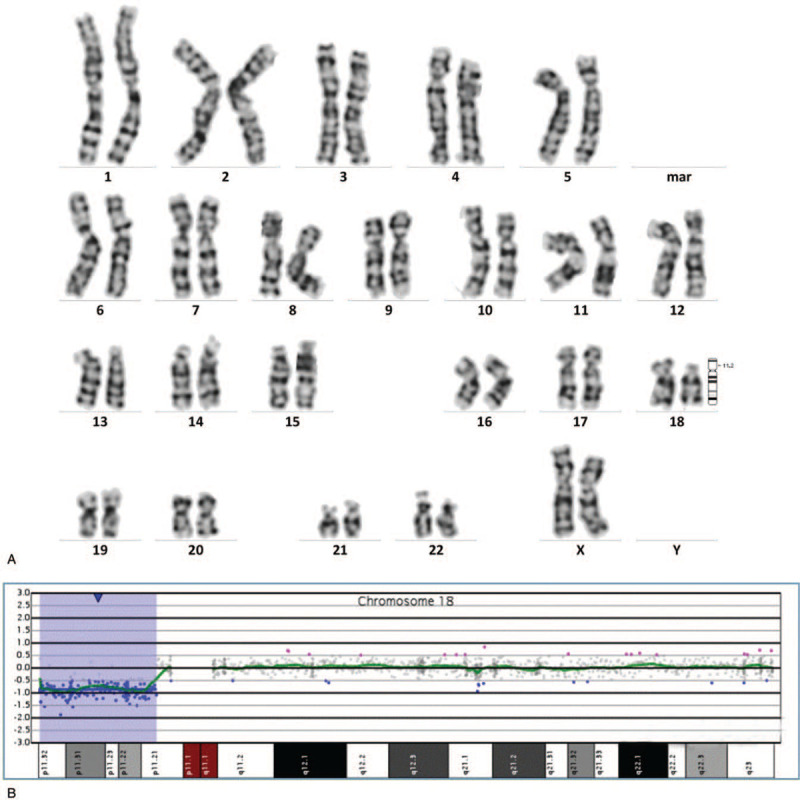
Prenatal diagnosis of 18p deletion syndrome in case 2. (A) The results of karyotype analysis: 46, XX, del (18)(p11.21). (B) The results of array-CGH:arr[GRCh37] 18p11.32p11.21 (146484-12532804)x1.

The karyotype analysis of the fetal amniotic fluid exfoliated cells was performed. The result of karyotype analysis showed an abnormal karyotype of 46, XX, del (18)(p11.21) (Fig. 2A). The array-CGH analysis results were arr[GRCh37] 18p11.32p11.21(146484_12532804)x1, indicating that the fetus had a deletion of about 12.39 Mb on chromosome 18 p11.32-p11.21 (Fig. 2B). It contains 47 OMIM genes, including *USP14*, *THOC1*, *COLEC12*, *CETN1*, *CLUL1*, *TYMS*, *ENOSF1*, *YES1*, *ADCYAP1*, *NDC80*, *SMCHD1*, *EMILIN2*, *LPIN2*, *MYOM1*, *MYL12B*, *TGIF1*, *DLGAP1*, *AKAIN1*, *ZBTB14*, *EPB41L3*, *L3MBTL4*, *ARHGAP28*, *LAMA1*, *PTPRM*, *RAB12*, *GACAT2,MTCL1*, *NDUFV2*, *ANKRD12*, *TWSG1*, *RALBP1*, *PPP4R1*, *RAB31*, *TXNDC2*, *VAPA*, *APCDD1*, *NAPG*, *PIEZO2*, *GNAL*, *CHMP1B*, *MPPE1*, *IMPA2*, *CIDEA*, *TUBB6*, *AFG3L2*, *PRELID3A*, and *SPIRE1*. The pregnant woman and her husband refused to undergo further karyotype analysis or array-CGH analysis and opted to terminate the pregnancy.

#### 4.2.2 Case 3

**Figure 3:**
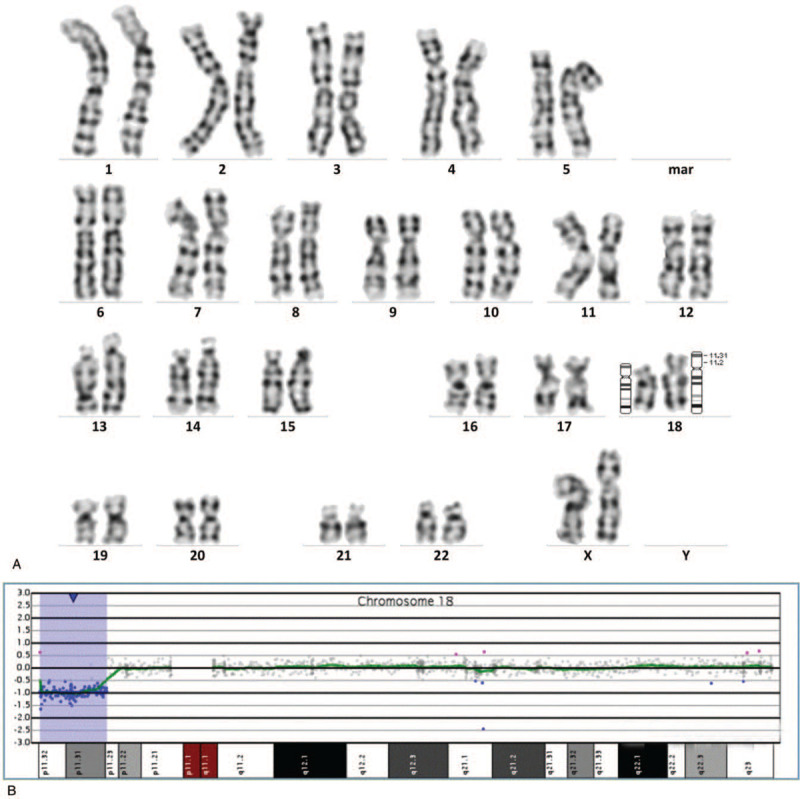
The results of prenatal diagnosis in case 3. (A) The results of karyotype analysis:46,XX,del (18)(?p11.2). (B) The results of array-CGH:arr[GRCh37] 18p11.32p11.23 (146484_7244642)x1.

The ultrasound-guided amniocentesis was performed on this pregnant woman. The array-CGH analysis results of the fetus were arr[GRCh37] 18p11.32p11.23(146484_7244642), which showed a deletion of about 7.1 Mb in 18p11.32-p11.23 (Fig. 3B) and consistency with the NIPT results. The missing fragment contains 23 OMIM genes, including *USP14*, *THOC1*, *COLEC12*, *CETN1*, *CLUL1*, *TYMS*, *ENOSF1*, *YES1*, *ADCYAP1*, *NDC80*, *SMCHD1*, *EMILIN2*, *LPIN2*,*MYOM1*, *MYL12B*, *TGIF1*, *DLGAP1*, *AKAIN1*, *ZBTB14*, *EPB41L3*, *L3MBTL4*, *ARHGAP28*, and *LAMA1*. The karyotype analysis did not give a precise result, and the chromosome karyotype was 46,XX,del(18)(?p11.2) (Fig. 3A). This microdeletion was not detected accurately with the traditional karyotype analysis, which might be attributable to the low resolution of G-banding. We recommended the couple to undergo array-CGH analysis. Unfortunately, the couple refused to undergo further detection and opted to terminate the pregnancy.

#### 4.2.3 Case4

**Figure 4:**
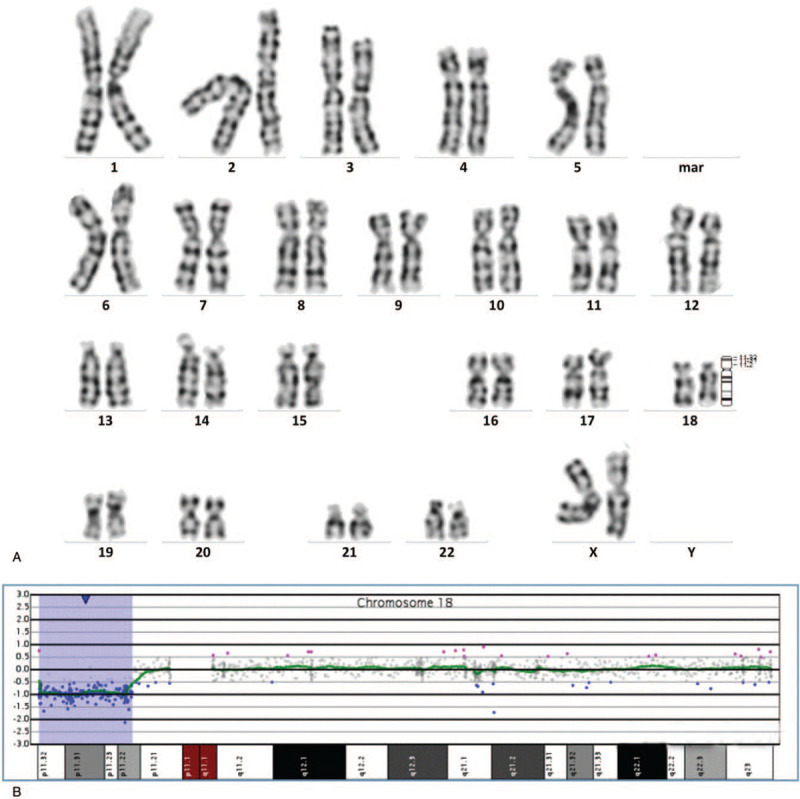
The results of prenatal diagnosis in case 4. (A) The results of karyotype analysis: 46, XX, del (18)(p11.2). (B) The results of array-CGH: arr[GRCh37] 18p11.32p11.22 (146484_10048312)x1.

The pregnant woman underwent amniocentesis at 19 + 2 weeks’ gestation. The result of the amniotic fluid karyotype analysis showed 46, XX, del (18)(p11.2) (Fig. 4A). The array-CGH analysis results were del(18)(p11.32p11.22), which indicated that the fetus had a deletion of about 9.9 Mb on chromosome 18 p11.32-p11.22 in size (146484_10048312) (Fig. 4B). The missing fragment contains 35 OMIM genes, including *USP14*, *THOC1*, *COLEC12*, *CETN1*, *CLUL1*, *TYMS*, *ENOSF1*, *YES1*, *ADCYAP1*, *NDC80*, *SMCHD1*, *EMILIN2*, *LPIN2*, *MYOM1*, *MYL12B*, *TGIF1*, *DLGAP1*, *AKAIN1*, *ZBTB14*, *EPB41L3*, *L3MBTL4*, *ARHGAP28*, *LAMA1*, *PTPRM*, *RAB12*, *GACAT2*, *MTCL1*, *NDUFV2*, *ANKRD12*, *TWSG1*, *RALBP1*, *PPP4R1*, *RAB31*, *TXNDC2*, and *VAPA*. The peripheral blood was collected from the couple, and karyotype analysis was performed. The results of karyotype analysis showed there was no abnormality in the couple's chromosome, indicating that the deletion was a de novo mutation in the fetus. Finally, the couple opted to terminate the pregnancy.

## 5 Discussion

**Figure 5:**
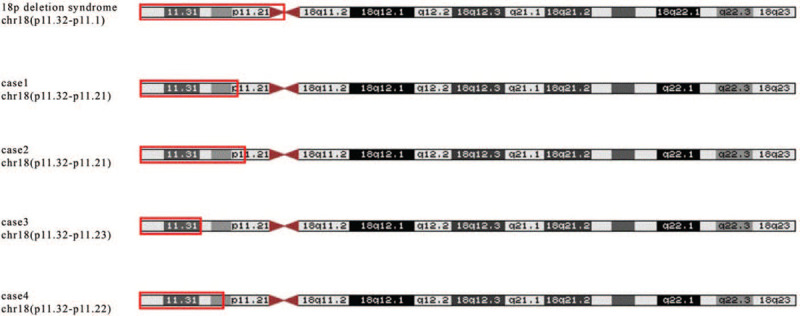
The chromosome breakpoints of 4 18p deletion syndrome cases.

The chromosome 18p deletion syndrome is first described in 1963 by de Grouchy et al., and more than 150 cases have been reported worldwide.^[1,2]^ A variety of clinical manifestations tend to be associated with the size and breakpoints of the deletion and the involved genes.^[7,8]^ A great deal of work has been done to determine the relationship between genotype and phenotype, but the genotype–phenotype correlation in this syndrome remains a challenge. In our report, we described 4 China cases with different chromosomal breakpoints (Fig. 5). We hope that our report can provide assistance for the diagnosis and the genetic counseling of the chromosome 18p deletion syndrome.

The 18p deletion syndromes are described well in the literature. About 2/3 of the cases of the 18p deletion syndrome result from de novo deletions, and the remainder is caused by the unbalanced transmission of structural rearrangements due to familial balance translocation, unbalanced whole-arm translocation between chromosome 18 and one of the acrocentric chromosomes, or familial transmission.^[9–12]^ To our knowledge, the familial transmission of the chromosome 18p deletion syndrome is very rare, and no >10 cases were reported.^[13–20]^ Almost all of the familial transmissions of the 18p deletion syndrome were delivered from a mother to a child, and the phenotypes of the 2 generations were similar.

In our report, the 18p deletion syndrome is transmitted from the woman to her fetus in case 1. This demonstrates that female patients with the 18p deletion syndrome have normal fertility, which is consistent with the findings in the literature. However, the woman who with mild phenotypes gave birth to a severely deformed fetus, it is different from previous reports. To our knowledge, we are the first to report that mother and progeny who have the same chromosome breakpoint have remarkably different phenotypes. This result may be attributed to our inability to accurately detect the molecular difference (such as point mutations in some genes). However, further research is needed in the future.

No good treatment is available for the 18p deletion syndrome and can only be prevented by prenatal diagnosis. Given the varied and untypical clinical presentation of the 18p deletion syndrome, a challenge remains in the prenatal diagnosis of this syndrome. Reports on the prenatal diagnosis of this syndrome are rare. The high risk of maternal serum screening may be useful for the screening of the 18p deletion syndrome.^[21]^ The INT or holoprosencephaly in prenatal ultrasound examination may also indicate the development of the 18p deletion syndrome.^[22–25]^ In our report, INT are found at 12 + 2 weeks of gestation in case 4, further assisting the diagnosis of the 18p deletion syndrome. In case 1, the fetus with holoprosencephaly was diagnosed as the 18p deletion syndrome. We have indicated that INT and holoprosencephaly can be used as ultrasound characteristics in the prenatal diagnosis of the 18p deletion syndrome.

NIPT is a noninvasive method used to screen abnormalities of the common fetal chromosome aneuploidy. The common fetal chromosome aneuploidy includes trisomy 21, trisomy 18, and trisomy 13. NIPT is highly sensitive on these 3 chromosomes and widely used in prenatal screening.^[26]^ Gan et al^[27]^ have reported the first case of prenatal diagnosis of the 18p deletion syndrome following NIPT, but the result of NIPT suggests that a partial or complete deletion of the X chromosome in their case. In our case 3, the fetus is also diagnosed as 18p deletion syndrome after NIPT, and our NIPT result accurately indicates the deletion on chromosome 18p. We believe that the sensitivity and specificity of NIPT are increasing, it may be used in prenatal screening of 18p deletion syndrome in the future.

In case 2, the ultrasonography suggests severe hydronephrosis in the fetus, and the final diagnosis was the 18p deletion syndrome. To our knowledge, this is the first case of prenatal diagnosis of the 18p deletion syndrome with hydronephrosis. Thus, we strongly suggest the pregnant woman to undergo a prenatal diagnosis when the ultrasonic examination indicates an abnormal renal development. Fetal hydronephrosis may be as a new phenotype of chromosome 18p deletion syndrome in prenatal.

## 6 Conclusions

In summary, the clinical phenotypes of the 18p deletion syndrome are varied and untypical. Our 3 prenatal cases proved that the phenotypes of the 18p deletion syndrome are differ among individuals with different chromosome breakpoints, which are consistent with previous reports. In addition, we found a familial transmission of 18p deletion syndrome case. The mother and fetus have an identical chromosomal deletion, but the phenotype of them represented different significantly. This finding is different from previous familial transmission reports of the 18p deletion syndrome. Therefore, we demonstrate that the individuals with different chromosomal breakpoints of 18p deletion syndrome have different phenotypes. On the other hand, the individuals with the same chromosomal breakpoints of 18p deletion syndrome may also have remarkably different phenotypes.

## Acknowledgments

The authors thank the subjects and families for participating in the study.

## Author contributions

**Conceptualization:** Shuai Zhen.

**Data curation:** Rong Qiang, Xiaobin Wang, Na Cai, Wen Zhai.

**Methodology:** Qiujie Jin, Rong Qiang, Bo Cai, Wen Zhai.

**Software:** Xiaobin Wang.

**Validation:** Na Cai.

**Writing – original draft:** Qiujie Jin.

**Writing – review & editing:** Qiujie Jin, Rong Qiang, Wen Zhai.
